# Cardamom Extract Alleviates the Oxidative Stress, Inflammation and Apoptosis Induced during Acetaminophen-Induced Hepatic Toxicity via Modulating Nrf2/HO-1/NQO-1 Pathway

**DOI:** 10.3390/cimb44110365

**Published:** 2022-11-02

**Authors:** Essraa A. R. Alkhalifah, Amjad A. Alobaid, Marwah A. Almajed, Manar K. Alomair, Lama S. Alabduladheem, Sarah F. Al-Subaie, Abdullah Akbar, Mahesh V. Attimarad, Nancy S. Younis, Maged E. Mohamed

**Affiliations:** 1 Department of Pharmaceutical Sciences, College of Clinical Pharmacy, King Faisal University, Al-Hofuf 31982, Saudi Arabia; 2 Reference Laboratory for Food Chemistry, Saudi Food and Drug Authority (SFDA), Riyadh 11561, Saudi Arabia

**Keywords:** acetaminophen, cardamom, hepatic function, inflammation

## Abstract

Acetaminophen (APAP) is the most extensively used and safest analgesic and antipyretic drug worldwide; however, its toxicity is associated with life-threatening acute liver failure. Cardamom (CARD), a sweet, aromatic, commonly used spice, has several pharmacological actions. In the current study, we tried to explore the chemical composition and the hepato-protective effect of ethanolic aqueous extract of CARD to mitigate APAP-induced hepatic toxicity and elucidate its underlying mechanism of action. Material and methods: Aqueous CARD extract was subjected to LC-TOF-MS analysis to separate and elucidate some of its components. In vivo animal experiments involved five groups of animals. In the normal and cardamom groups, mice were administered either saline or CARD (200 mg/kg), respectively, orally daily for 16 days. In the APAP group, the animals were administered saline orally daily for 15 days, and on the 16th day, animals were administered APAP (300 mg/kg) IP for the induction of acute hepatic failure. In the CARD 200 + APAP group, mice were administered CARD (200 mg/kg) for 15 days, followed by APAP on the 16th day. Results: The aqueous extract of CARD showed several compounds, belonging to polyphenol, flavonoids, cinnamic acid derivatives and essential oil components. In the in vivo investigations, APAP-induced impaired liver function, several histopathological alterations, oxidative stress and inflammatory and apoptotic status signified severe hepatic failure. Whereas, pretreatment with the CARD extract prior to APAP administration diminished serum levels of the hepatic function test and augmented Nrf2 nucleoprotein and HO-1 and NQO-1. CARD down-regulated MDA, inflammatory mediators (IL-1β, IL-6, TNF-α and NF-κB) and apoptotic markers (caspase 3 and 9 and Bax) and amplified the activities of SOD, catalase, GSH-Px and GSH-R in hepatic tissue samples. Conclusion: CARD extract mitigated the hepatic toxicity induced by APAP. The underlying mechanism of action of such hepato-protective action may be through upregulation of the Nrf2/HO-1/NQO-1 pathway with subsequent alleviation of the oxidative stress, inflammation and apoptosis induced by APAP. Many of the compounds identified in the CARD extract could be attributed to this pharmacological action of the extract.

## 1. Introduction

Acute liver failure (ALF) is an emergent threat to human health globally. ALF is initiated by numerous influences, such as hepatitis viral infection, toxin exposure and drug overdoses. Acetaminophen or paracetamol (APAP) is an analgesic and antipyretic drug that is broadly used to treat different conditions associated with pain and fever [1]. It is incorporated in both prescribed and over-the-counter drugs with different concentrations [2]. APAP is generally considered a safe drug at normal therapeutic dose levels; however, its major health hazard is liver failure in the acute overdose [3]. APAP toxicity is related to its metabolism to N-acetyl-p-benzoquinone imine (NAPQI) via cytochrome P450 [4]. NAPQI depletes GSH with subsequent generating of ROS, leading to oxidative stress inflammation and liver injury. In addition, NAPQI is able to prompt protein impairment, mitochondrial dysfunction, ATP exhaustion, nitrogen species synthesis and lipid peroxidation (LPO), causing hepatocytes to undergo necrosis, apoptosis and cellular destruction [5]. APAP overdose-induced oxidative stress initiates diverse signal pathways, such as nuclear factor-kappa B (NF-κB), which up-regulates different pro-inflammatory cytokines and inflammatory mediators, comprising TNF-α, IL-1β and cyclooxygenase-2 (COX2) [6]. 

Oxidative stress is the main player in liver pathology and its processes [7]. Hepatocellular necrosis deteriorates with a decrease in the activity of antioxidative enzymes, such as catalase, superoxide dismutase (SOD) or glutathione peroxidase (GPx) [8]. The nuclear factor erythroid 2–related factor 2 (Nrf2) is a crucial controller of the antioxidant defense system, which mediates cell survival as well as adjusts the gene expression encoding intracellular detoxifying enzymes and antioxidant proteins via antioxidant response element (ARE) [8,9]. Nrf2-dependent ARE-driven genes encoding detoxification and antioxidant enzymes include NAD(P)H: quinone oxidoreductase 1 (NQO1) and heme oxygenase-1 (HO-1) [10]. Previous studies have shown that Nrf2 is a possible target for the management of APAP-induced toxicity [11]. Therefore, Nrf2 modulation and/or anti-oxidative stress is an efficient way to limit or even avert acute liver failure initiated by APAP overdose. Natural compounds have been widely utilized as detoxification drugs due to their rich diversity, limited adverse actions and protective potential. 

Cardamom (CARD), the queen of spices, is a sweet, aromatic, commonly used spice and consists of entire or powdered dried fruit of *Elettaria cardamomum* L. (Maton), family Zingiberaceae [12]. Its dried fruit is used as a flavoring agent and in medical preparations. In addition, CARD has been used in traditional medicine to treat throat infections, high blood pressure, kidney disorders and some cardiovascular diseases [13]. In humans, a double blind randomized clinical trial examined the effect of 10-week green CARD intake on blood pressure, concentrations of inflammatory and endothelial function biomarkers in type 2 diabetes mellitus (T2DM) patients, and its potential mechanisms. CARD reduced HbA1c, insulin level, HOMA-IR and TG level via proliferating serum sirtuin-1 (SIRT1) concentration in T2DM patients [14,15]. CARD parts displayed several pharmacological potentials. For instance, CARD in basal diet prohibited hepatic and cardiac injury induced via radiation by ameliorating radiation induced oxidative stress [16]. CARD powder supplementation mitigated dyslipidemia, oxidative stress and hepatic damage in high carbohydrate and high fat (HCHF) diet-fed rats [17]. CARD oil protected against chemically induced hepatocellular carcinoma [18] and against Uranium hazards [19]. The aqueous CARD extract showed vasorelaxant [20], antibacterial [21], antioxidant [20,22], anti-inflammatory [23], immunomodulatory and anti-cancer [24] and macrophages modulator [25] potentials. Furthermore, aqueous extract of CARD attenuated doxorubicin (DOX)-induced cardiac toxicity [26], protected against isoproterenol-induced myocardial infarction [27] and prevented L-NAME-induced hypertensive [20]. The methanolic extract of CARD protected against ethanol-induced hepatotoxicity [22]. 

Although the pharmacological activities are widely investigated, the aqueous or ethanolic extracts of CARD are hardly studied chemically. Rahman, et al. [17] identified the presence of five phenolic compounds in the aqueous ethanolic extract of cardamom. Faiza, et al. [28] showed the presence of some phenolic compounds, such as gallic acid, ferulic acid and ellagic acid, in the aqueous extract. Ashokkumar, et al. [29] reviewed the phytochemistry of cardamom and illustrated the presence of many classes of compounds such as flavonoids, carotenoids and terpenoids.

Little is recognized as to whether CARD can be used as a hepatoprotective adjuvant to limit APAP-induced liver toxicity. Thus, in the current study, we tried to explore the hepato-protective ability of CARD to alleviate the hepatic toxicity induced by APAP and elucidate its underlying mechanism of action.

## 2. Materials and Methods

### 2.1. Preparation of CARD Aqueous Extract 

CARD fruits were purchased from the local market of agricultural herbs, spices and medicinal plants in Al Hasa, Saudi Arabia. The fruit was identified by Dr. Hesham Abdel-All, Professor of Medicinal and Aromatic Plants, Horticulture Department, College of Agriculture, Zagazig University. The plant was identified as *Elettaria cardamomum* L. (Maton), family Zingiberaceae. Voucher specimens (No. ZG544) were deposited in the herbarium of the College of Clinical Pharmacy, King Faisal University, Saudi Arabia.

Following the grinding of the fruits and seeds, the resultant powder (50 g) was mixed thoroughly with distilled water (200 mL) with stirring (150 RPM) for 6 h. The obtained solution was filtered twice through a Whatman filter No.1 and finally centrifuged at 5000 rpm for 15 min. This extraction process was repeated two more times on the mark left after decantation, filtration and centrifugation. All the filtrate was poured together and concentrated under reduced pressure to obtain a residue. The residue was adjusted by saline to reach a final concentration of 100 mg/mL (*w*/*v*) [21,26]. 

### 2.2. Liquid Chromatography Time-of-Flight Mass Spectrometry (LC-TOF-MS) Analysis

Analyses were conducted using Agilent 1290 series LC systems (Santa Clara, CA, USA) equipped with a Binary Solvent Manager, Sample Manager—Flow-Through-Needle, and a column heater with active pre-heating, coupled with an AB SCIEX QTOF mass spectrometer with a roughing pump. A Gemini-C18 column (150 × 4.6) mm 3-μm particle diameter (Phenomenex, Torrance, CA, USA) was used for column separation. The column temperature was maintained at 30 °C; the flow rate was 0.4 mL/min; and the injection volume was 10 µL. The optimal mobile phase consisted of a linear gradient system of mobile phase (A) 0.3% Formic acid in water and (B) 0.3% Formic acid in (60% ACN and 40% MeOH). The linear gradient elution was automated in 20 min starting with 100% of mobile phase A with gradual increase of organic phase until ending with 100% of mobile phase B in 18 min with 2 min for washing as follows: 100% solvent A (minutes 0 to 1); 100% Solvent A to 100% solvent B (minutes 1 to 15); 100% solvent B (minutes 15 to 18); 100% Solvent B to 100% solvent A (minutes 18–19); and 100% solvent A (minute 20) 

The positive and negative mode was applied in the ESI source with the following parameters: gas 1 = 60 psi; gas 2 = 60 psi; temperature = 550 °C; and (5500 V in positive and −4500 V in negative) for ion spray voltage with 30 psi curtain gas. Intact protonated molecular ions [M-H] + and [M-H] − were detected via QTOF–MS scan (80 psi declustering potential, 10 V collision energy, 50–950 Da QTOF MS scan range and 150 ms accumulation time). The MS data were imported into MZmine 2.53 software for mass detection, chromatogram building, deconvolution, alignment, gap filling, R.T. normalization and identification using 3 online databases (PubChem, KEEG and MetaCyc). The data were exported into a csv file.

### 2.3. Radical Scavenging Properties of Cardamom 

Antioxidant activity of ethanolic extract was determined by free radical scavenging activity by DPPH method according to the procedure established by Blois [30] with slight modification. The sufficient amount of sample solutions were transferred to volumetric flasks to get the final concentrations of 500, 400, 300, 200 and 100 µg/mL in total volume of 4 mL. One ml of a 0.3 mM DPPH solution and sufficient amount of ethanol were added to the above sample solution of different concentrations to get a total volume of 4 mL. The volumetric flasks were covered with aluminum foil, and after 30 min, the absorbance values were measured at 517 nm and converted to the percentage antioxidant activity (AA) using the following formula:

AA% = 100 − ((Absorbance of sample − Absorbance of blank) × 100/Absorbance of control)

1 mL CARD extract solution in 4 mL ethanol was utilized as a blank solution. A control solution was performed using ethanol (3 mL) and DPPH solution (1 mL:0.3 mM). The EC50 values were calculated by linear regression of plots, where the abscissa represented the concentration of tested CARD extract and ordinate the average percent of antioxidant activity.

### 2.4. Animal’s Acquisition and Ethical Statement 

Male BALB/c mice with an average weight of 28 g (6–8 weeks old) were obtained from the Department of Biological Sciences, College of Science, King Faisal University, Saudi Arabia. They were maintained as six mice per each ventilated cage at the College of Medicine Laboratory Animal Center. The animals were sustained with standard laboratory food and water ad libitum in a system of ventilated cages (12 h light/dark cycles, 20.3–23.1 °C) throughout the whole experiment. The experimental protocol was permitted by the Institutional Animal Care and Use Committee of King Faisal University (KFU-REC-2022-APR-EA000592). All the experiments were executed in harmony with the relevant procedures and regulations of the Ethical Conduct for the Use of Animals in Research at King Faisal University.

### 2.5. Experimental Design 

Animals (*n* = 6) were arbitrarily allocated into four groups. In the normal and CARD groups, mice were administered saline or CARD (200 mg/kg) respectively, orally daily for 16 days. In the APAP group, the animals were administered saline orally daily for 15 days, and on the 16th day, animals were administered APAP (300 mg/kg) IP for the induction of acute hepatic failure [31,32]. In the CARD 200 + APAP group, mice were administered CARD (200 mg/kg) for 15 days, followed by APAP (300 mg/kg) IP. CARD dose selection was according to previously mentioned studies by [27]. The mice were anesthetized and sacrificed 3 h after APAP injection. Blood was collected through cardiac puncture and centrifuged to obtain serum for further biochemical analysis. Hepatic tissues were collected for histological and molecular analyses.

### 2.6. Histopathological Investigation 

For hepatic histological, a portion of mouse hepatic tissues was fixed with 10% formalin and processed; sections (4 μm) were obtained and stained with hematoxylin and eosin (H&E). Digital images were collected under a light microscope at 100× magnification. The histological changes were quantified as normal, moderate and severe based on the hepatic cytoplasm inflammation, centrilobular necrosis, cellular hypertrophy, vacuolization and steatosis [33]. The histopathological changes were quantified by two independent histopathologists and scored double blindly

### 2.7. Hepatic Function Tests Determination 

Colorimetric kits were used to determine hepatic function tests including alanine aminotransferase (ALT), aspartate aminotransferase (AST), alkaline phosphatase (ALP) and lactate dehydrogenase (LDH) following the manufacturer’s instructions.

### 2.8. Hepatic Oxidative Stress Status Determination 

Malondialdehyde (MDA; ab238537), glutathione peroxidase (GSH-Px; ab102530) and glutathione reductase (GSH-R; ab83461) ELISA kits were acquired from Abcam Inc. (Cambridge, UK). Superoxide dismutase (SOD; MBS036924) and catalase (MBS726781) ELISA kits were obtained from My BioSource (San Diego, CA, USA). All the procedures were executed in agreement with the manufacturer’s directions. 

### 2.9. Determination of Inflammation and Apoptotic Signaling Markers

Inflammation markers including TNF-α (ab46070), IL-1β (ab100768), IL-6 (ab100772) and IL-10 (ab133112) ELISA kits were obtained from Abcam Co., Eugene, OR, USA. As for the apoptotic signaling markers, cleaved caspase-3 (KHO1091) was purchased from Thermo Fisher Scientific Inc., Waltham, MA, USA, whereas caspase-9 (LS-F4141) was acquired from Biocompare, San Francisco, CA, USA. These markers were measured according to the manufacturer’s instructions using a microplate reader SpectraMax i3X (Molecular devices, San Jose, CA, USA). 

### 2.10. Gene Expression Experiments (Real-Time PCR) 

Real-time PCR was performed according to the technique described elsewhere [25]. Briefly, the Trizol reagent kit (Invitrogen, Waltham, MA, USA) and reverse transcription-polymerase chain reaction (RT-PCR) kit (TaKaRa, (Shiga, Japan), Cat. No. RR037A) were used to cleanse total RNA and inverse transcription reaction, respectively. In total, 20 μL of the reaction volume was mixed with 1 μL total RNA (1 μg/μL), incubated at 42 °C for 15 min, followed by 95 °C for 2 min, and the generated cDNA was stored at −20 °C. In total, 50 μL of PCR reaction mixture enclosed × 50 ROX Reference Dye (1 μL), sense and antisense primers (1 μL each, primers are mentioned in Table 1), ×2 SYBR Green PCR Master Mix (25 μL), cDNA template (4 μL) and sterilized distilled H2O (18 μL). The PCR reaction condition incorporated pre-denaturing at 95 °C for 10 s, then 40 cycles of 95 °C/5 s and 60 °C/30 s, and 72 °C/1 min. Quantification analyses were completed via Opticon-2 Real-Time PCR reactor (MJ Research, Reno, NV, USA). Step PE Applied Biosystems (Perkin Elmer, Waltham, MA, USA) analyzed real-time PCR results. Expression of the target gene was measured and correlated to the reference gene (β-actin). β-actin expression was used for sample normalization, where the 2 − ΔΔCT equation was used for relative expression determination. 

The primers used are as follows Nrf2 (NM_031789.2) F: 5′-CATTTGTAGATGACCATGAGTC GC-3′, R: 3′-ATCAGGG GTGGTGAAGACTG-5′; HO-1 (NM_012580.2) F: 5′-GTGCACATC CGTGCAG AGAA-3′, R: 3′-GTGCACAT CCGTGCAGAGAA-5′; NQO-1 (NM_008706) F: 5′-GTCCATTC-CAGCTGACAACCA-3′, R: 3′-GTCCATTCCAGCTGAC AACCA-5′; NFκB F: 5′-TGGGACGACACCTCTACACA-3′, R: 3′-GGAGCTCATCTCAT AGTTGTCC-5′; Bcl-2 (NM_016993.1) F: 5′-CCGGGAGATCGTGATGAAGT-3′, R: 3′-ATC CCAGCCTCCGTTATC CT-5′; Bax (NM_017 059.2) F: 5′-GTGGT TGCCCTCTTCTACTT TG-3′, R: 3′-CACAAAGA TGGTCACTGTCTGC-5′; β-actin (NM_0 3144.3) F: 5′-TGACA GGATGCAGAAGGAGA-3′, R: 3′-TA GAGCCACCA ATCCACACA-5′.

### 2.11. Statistical Analysis

Data are presented as mean ± SD. For multiple comparisons, one-way ANOVA followed by Tukey–Kramer as a post hoc test was performed. The 0.05 level of probability was used as the significance level. All statistical analyses were performed using Graph Pad software (version 5, San Diego, CA, USA).

## 3. Results

### 3.1. LC-TOF-MS Analysis of CARD Extract

**Figure 1:**
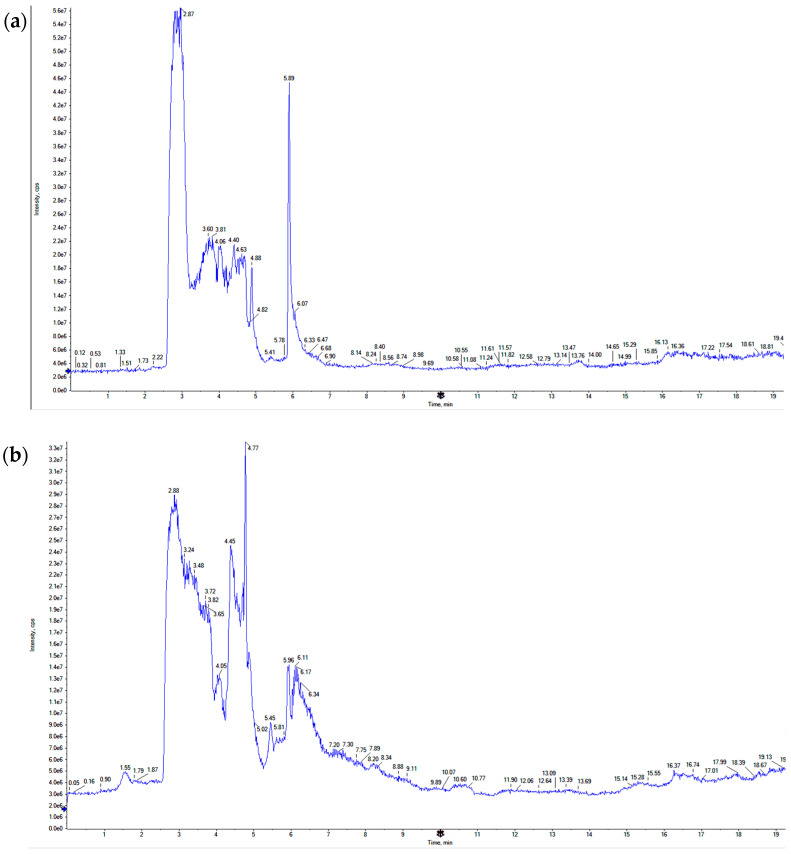
LC-TOF-MS analysis of CARD aqueous extract. (**a**) Positive mode MS chromatogram. (**b**) Negative mode MS chromatogram.

The aqueous extract of CARD was subjected to LC-TOF-MS separation (Figure 1), followed by compound identification using compound retention times and mass fragmentation patterns in relation to several online libraries (Table 1). Several compounds were separated from the extract; however, only 25 compounds were identified, representing 68.229% of the total separated compounds. Many classes of compounds were identified in CARD extract, including Flavonoids (32.087%), polyphenols and phenolic acids (45.862%), cinnamic acid derivatives (15.448%) and essential oil components (4.839%). The most abundant compounds separated were identified, having Luteolin and gallic acid as the most abundant (9.275% and 8.180 %, respectively). Many other compounds represent concentrations more or around 4%, such as catechin (4.295%), ferulic acid (3.972%), quercetin (5.372%), *trans*-cinnamic acid (4.188%) and 8-methoxyquercetin (4.654%). 

### 3.2. DPPH Assay of CARD Extract 

**Figure 2:**
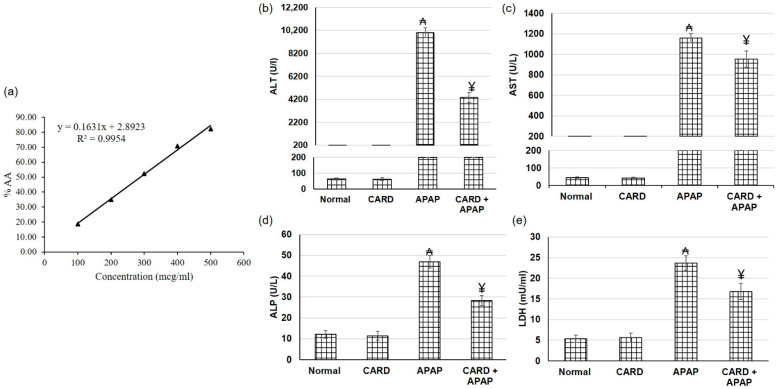
Effects of CARD aqueous (200 mg/kg) extract administration for 15 days prior to APAP administration (300 mg/kg) (**a**) DPPH and on the hepatic function tests, including (**b**) ALT, (**c**) AST, (**d**) ALP and (**e**) LDH in APAP induced acute hepatic failure in mice. All values are stated as mean ± SD. ₳ designates statistically significant compared to the normal group and ¥ designates statistically significant compared to the APAP group (*p* < 0.05) using one-way ANOVA followed by Tukey’s post hoc test.

DPPH method has been extensively used for the determination of antioxidant activity by the free radical scavenging effect of the plant extract. Free radical scavengers donate the proton to DPPH and convert it to the DPPHH, which decreases the violet color of the DPPH. The CARD extract showed good antioxidant activity with EC_50_ of 290.8 µg/mL, as illustrated in Figure 2a.

### 3.3. CARD Extract Alleviated APAP-Induced Acute Hepatic Failure 

Hepatic function tests, including ALT, AST, ALP and LDH, were used to assess the hepatic function. In the APAP-induced acute hepatic failure group, ALT, AST, ALP and LDH serum levels were significantly amplified, demonstrating impaired liver function (Figure 2). On the other hand, pretreatment with the aqueous extract of cardamom prior to APAP administration diminished ALT, AST, ALP and LDH serum levels significantly compared to APAP alone group, which indicated that cardamom extract might exert a protective effect against APAP-induced hepatic injury.

### 3.4. CARD Mitigated APAP-Induced Acute Hepatic Failure Alterations in Histopathological Examination

**Figure 3:**
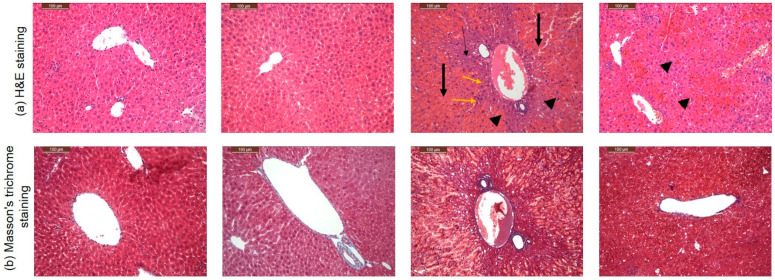
Effects of CARD aqueous (200 mg/kg) extract administration for 15 days prior to APAP administration (300 mg/kg) on histopathological changes in the hepatic sections stained with (**a**) hematoxylin and eosin (H&E) and (**b**) Masson’s trichome staining in APAP induced acute hepatic failure in mice.

Consistent with the liver function tests, mice haptic tissue obtained from control and CARD groups, stained with H&E, showed normal architectures of hepatic parenchyma. However, APAP haptic samples exhibited massive necrosis (Figure 3, thick arrows) with a few zones of surviving hepatocytes around the central vein (Figure 3, headed arrow), mild hydropic degeneration (Figure 3, yellow arrows), central vein and hepatic sinusoids congestion, and hemorrhage. CARD + APAP group showed restoring of hepatic damage as characterized by binucleated hepatocytes (Figure 3, head arrows) with mild congestion.

### 3.5. CARD Amplified Nrf2/HO-1/NQO-1/NF-κB Pathway Gene Expression in APAP-Induced Acute Hepatic Failure 

**Figure 4:**
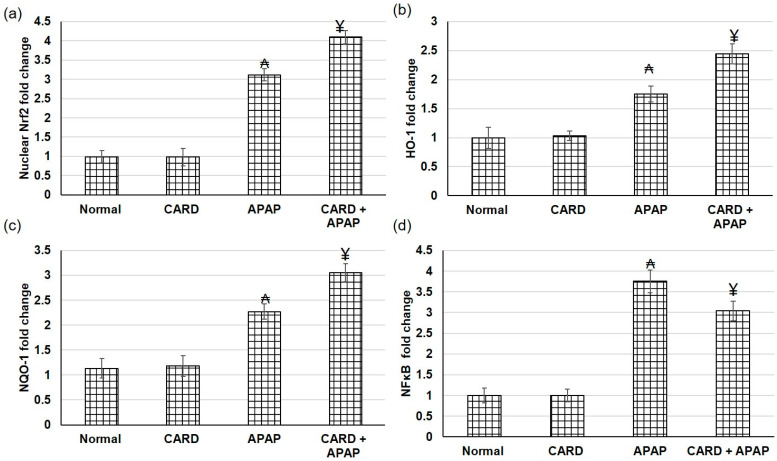
Effects of CARD aqueous extract (200 mg/kg) administration for 15 days prior to APAP administration (300 mg/kg) on gene (mRNA) expression levels of (**a**) Nrf2, (**b**) HO-1, (**c**) NQO-1 and (**d**) NF-κB, respectively, in APAP-induced acute hepatic failure in mice. All values are stated as mean ± SD. ₳ designates statistically significant compared to the normal group, and ¥ designates statistically significant compared to the APAP group (*p* < 0.05) using one-way ANOVA followed by Tukey’s post hoc test.

Real-time PCR was implemented to identify the gene expression level of the Nrf2/HO-1/NQO-1 pathway. Compared to the normal and CARD groups, the gene expression levels of Nrf2 nucleoprotein and total HO-1 and NQO-1 were considerably augmented in the APAP-induced acute hepatic failure, as presented in Figure 4. On the other hand, gene expression levels of Nrf2 nucleoprotein and total HO-1 and NQO-1 were further augmented, whereas NF-κB was decreased in the animals pretreated with cardamom compared to APAP alone animals. 

### 3.6. CARD Alleviated Oxidative Stress in APAP-Induced Acute Hepatic Failure 

**Figure 5:**
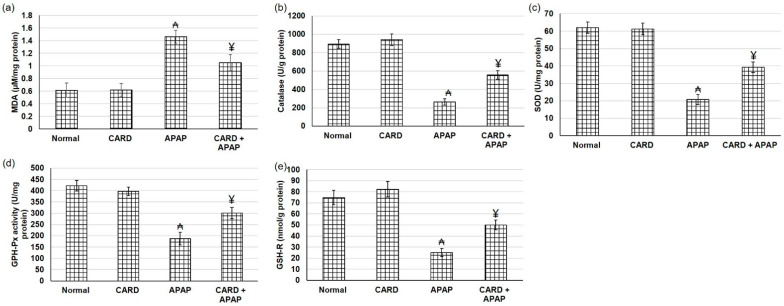
Effects of CARD aqueous extract (200 mg/kg) administration for 15 days prior to APAP administration (300 mg/kg) on lipid peroxidation, including (**a**) MDA content and antioxidant enzymes activities, including (**b**) catalase, (**c**) superoxide dismutase (SOD), (**d**) glutathione peroxidase (GSH-Px) and (**e**) glutathione reductase (GSH-R) activities, respectively, in APAP-induced acute hepatic failure in mice. All values are stated as mean ± SD. ₳ designates statistically significant compared to the normal group, and ¥ designates statistically significant compared to the APAP group (*p* < 0.05) using one-way ANOVA followed by Tukey’s post hoc test.

Compared to the normal group, APAP tissue samples showed markedly greater levels of MDA content, indicating lipid peroxidation elevation, which was down-regulated with Cardamom (200 mg/kg) pretreatment (Figure 5a). In contrast to MDA, hepatic APAP tissue samples exhibited lowered levels of antioxidant enzyme activities, including SOD, catalase, GSH-Px and GSH-R. Whereas pretreatment with aqueous extract of cardamom amplified the activities of SOD, catalase, GSH-Px and GSH-R in hepatic tissue samples when related to APAP alone (Figure 5b–e).

### 3.7. CARD Alleviated Hepatic Inflammation and Apoptosis Responses in APAP-Induced Acute Hepatic Failure

**Figure 6:**
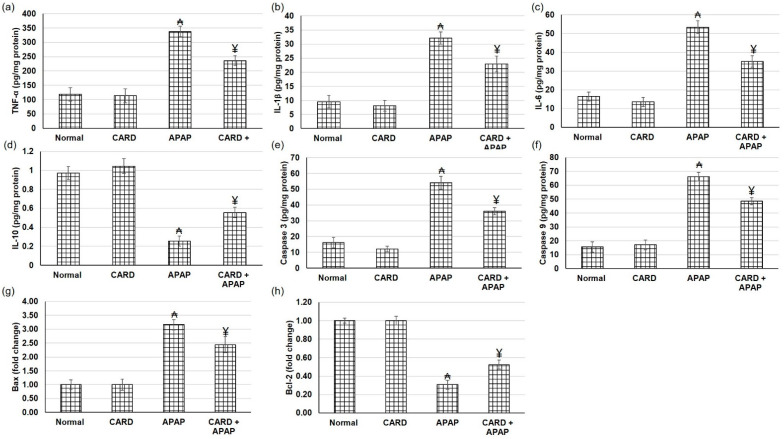
Effects of CARD aqueous extract (200 mg/kg) administration for 15 days prior to APAP administration (300 mg/kg) on the levels of (**a**) TNF-α, (**b**) IL-1β, (**c**) IL-6, (**d**) IL-10, (**e**) caspase 3, (**f**) caspase 9 and gene expression of (**g**) Bax, and (**h**) Bcl2, respectively, in APAP-induced acute hepatic failure in mice. All values are stated as mean ± SD. ₳ designates statistically significant compared to the normal group, and ¥ designates statistically significant compared to the APAP group (*p* < 0.05) using one-way ANOVA followed by Tukey’s post hoc test.

Figure 6 illustrates the escalation in the inflammatory mediators, including IL-1β, IL-6 and TNF-α, in the animals that experienced APAP when related to normal groups. These intensifications were deterred in animals treated with aqueous extract of cardamom (200 mg/kg), as demonstrated in Figure 6. As for the apoptosis response, animals administered APAP exhibited intensified apoptotic markers, including caspase 3 and 9 levels and Bax gene expression, whereas Bcl2 gene expression was diminished (Figure 6e–h). On the other hand, cardamom resulted in apoptosis mitigation as demonstrated by the lowered caspase 3 and 9 and Bax and elevated Bcl2 confirming the anti-apoptotic effect of CARD.

## 4. Discussion

CARD is a popular spice traditionally used in medicine because of its potent antioxidant and anti-inflammatory properties. Nevertheless, little is known about whether CARD can be used as a hepatoprotective adjuvant to limit APAP liver toxicity, and its underlying mechanism of action has not been elucidated yet. Collectively, the current study showed that pretreatment with CARD constrained the hepatic damage (including structural and functional disruptions) induced by APAP. These actions may be, at least in part, through the inhibition of ROS release and the reduction of inflammation and apoptotic marker. In the current study, APAP-induced impaired liver function and hepatic failure as demonstrated by the amplified hepatic function tests and the histopathological examination, which was demonstrated earlier by several reports [34,35]. Whereas, pretreatment with the aqueous extract of CARD prior to APAP administration diminished ALT, AST, ALP and LDH serum levels, demonstrating that CARD extract might exert a protective effect against APAP-induced hepatic injury. A previous study showed that methanol extract of CARD protected against ethanol-induced hepatotoxicity as reflected by the lowered level of liver enzymes and serum lipid profile [22]. Furthermore, CARD oil reduced the levels of hepatic malondialdehyde and liver injury markers, such as ALT, AST, ALP and γ-glutamyl transferase (GGT), in DENA-induced hepatocellular carcinoma [18]. In addition, CARD prevented radiation-induced liver and heart damage through which it decreased ALT, AST, ALP, LDH, TC, TAG, LDL-C and iron concentration [16]. 

Previous studies showed the significant role of Nrf2 in APAP-induced hepatic toxicity. For instance a study reported that Nrf2^−/−^ mice experienced greater hepatotoxicity and mortality [36] and increased sensitivity to APAP-induced liver injury [37,38]. In the existing study, the gene expression levels of Nrf2, HO-1 and NQO-1 were augmented in the APAP-induced acute hepatic failure, whereas pretreatment with CARD extract caused further augmented Nrf2, HO-1 and NQO-1. Similarly, oral administration of CARD to 7,12-dimethylbenz[a]anthracene (DMBA)-treated mice up-regulated the phase II detoxification enzymes, probably via activation of Nrf2 [13]. Nrf2 plays a major role in the defense against APAP toxicity through the GSH synthesis pathway [39]. Furthermore, The Nrf2^−/−^ mice had difficulty in detoxifying APAP and its metabolites because of the lower level of hepatic GSH [37]. In the existing study, APAP markedly increased MDA content levels, signifying lipid peroxidation escalation. Additionally, APAP depressed antioxidant enzyme activities, including superoxide dismutase (SOD), catalase, glutathione peroxidase (GSH-Px) and glutathione reductase (GSH-R). In comparison, pretreatment with aqueous extract of CARD amplified the activities of SOD, catalase, GSH-Px and GSH-R and down-regulated MDA content in hepatic tissue samples when related to APAP alone. Similarly, aqueous extract of CARD attenuated doxorubicin-induced cardiotoxicity via lowering NO, and MDA levels and up-regulating SOD, CAT and GPx level [26]. Besides, treatment with CARD aqueous extract prevented the exhaustion of GSH from the heart and inhibited lipid peroxidation (MDA content) in isoproterenol-induced myocardial infarction in rats [27]. CARD extract prevented vascular remodeling and oxidative stress parameters linked to hypertension induced by L-NAME. Another study showed that CARD oil also significantly decreased oxidative stress indicators in DENA-induced hepatocellular carcinoma [18]. In ethanol-induced hepatotoxic effect, methanol extract CARD reduced MDA and increased SOD, GSH-Rd activity [22]. While adding CARD to the basal diet before gamma radiation diminished oxidative stress markers in both liver and heart tissues [16]. 

In the existing study, an escalation in the inflammatory mediators, including IL-1β, IL-6, TNF-α and NF-κB, and apoptosis indicators, including caspase 3 and 9 levels and Bax gene expression, were demonstrated in the animals that were challenged with APAP. These intensifications were deterred in animals pretreated with aqueous extract of CARD prior to APAP. Aqueous extract of CARD exhibited anti-inflammatory and anti-apoptotic effects in various animal models. For instance, in lipopolysaccharide (LPS) induced inflammatory response, aqueous extracts of CARD exhibited a decrease in the gene expression of pro-inflammatory cytokines induced by LPS [23]. Furthermore, aqueous extracts of CARD showed anti-inflammatory and anti-apoptotic effects in the doxorubicin-induced cardiotoxicity [26] and the isoproterenol-induced myocardial infarction. Additionally, CARD oil decreased the levels of TNF-α, IL-1β and NF-κB in the DENA-induced hepatocellular carcinoma [18].

## 5. Conclusions

In conclusion, the CARD aqueous extract showed the presence of polyphenols and flavonoids as major compounds, with some cinnamic acid derivatives and essential oil components. The aqueous extract demonstrated in vitro antioxidant activity and it was able to mitigate the acute hepatic failure induced by APAP. The underlying mechanism of action of such hepato-protective action may be through the upregulation of the Nrf2/HO-1/NQO-1 pathway with subsequent alleviation of the oxidative stress, inflammation and apoptosis prompted by APAP. The novelty of this research relies on the discovery of pharmacological activity of CARD as a protective agent against oxidative stress-induced liver failure. This research could add to the pool of the pharmacological activities of CARD, especially the aqueous extract of the fruit, which represents one of the common and traditional ways to use CARD here in the Arab world, particularly with coffee, tea and other hot beverages.

## Figures and Tables

**Table 1:** Some of the chemical constituents identified in the aqueous extract of CARD after the LC-TOF-MS separation.

#	Retention Time (Minutes)	Compound Name	Peak Area Percentage
1	2.888	Gallic acid	8.18
2	2.904	Catechin	4.295
4	3.014	Ferulic acid	3.972
5	3.024	Quercetin	5.372
6	3.037	Luteolin	9.275
7	3.09	Cafeic acid	1.236
8	3.166	Abscisic alcohol	1.834
9	3.63	Trans-cinnamic acid	4.188
10	3.688	Davidigenin	1.124
11	3.762	Lunularic acid	2.706
12	3.949	Patuletin	1.547
13	3.984	Ellagic acid	1.765
14	4.091	8-methoxyquercetin	4.654
15	4.144	Apigeninidin	3.701
16	4.309	*trans*-Cinnamoyl beta-D-glucoside	1.094
17	4.532	Glutamyl-glutamic acid	3.583
18	5.388	Traumatic acid	0.493
20	5.895	Hydrocinnamamide	1.128
21	5.914	Myricetin	3.243
22	6.108	*cis*-Citral	1.007
23	16.539	(+)-Camphor	1.616
24	16.667	*trans*-Carveol	1.04
25	19.148	Limonene-1,2-epoxide	1.176
Total			68.229
Compound group name			Area percentage
Flavonoids			32.087
Polyphenols and phenolic acids			45.862
Cinnamic acid derivatives			15.448
Essential oil components			4.839

## Data Availability

Not applicable.
